# Beneficial Effects of Palmitoylethanolamide on Expressive Language, Cognition, and Behaviors in Autism: A Report of Two Cases

**DOI:** 10.1155/2015/325061

**Published:** 2015-09-29

**Authors:** Nicola Antonucci, Alessandra Cirillo, Dario Siniscalco

**Affiliations:** ^1^Biomedical Centre for Autism Research and Treatment, 70126 Bari, Italy; ^2^Institute of Biosciences and Bioresources, National Research Council of Italy, 80128 Naples, Italy; ^3^Department of Experimental Medicine, Second University of Naples, Via S. Maria di Costantinopoli, 80138 Naples, Italy; ^4^Centre for Autism-La Forza del Silenzio, 81036 Caserta, Italy; ^5^Cancellautismo-No Profit Association for Autism Care, 50132 Florence, Italy

## Abstract

*Introduction*. Autism spectrum disorders are defined by behavioral and language atypias. Growing body of evidence indicates inflammatory mediators may contribute to the condition. Palmitoylethanolamide (PEA) is naturally occurring and has been available as a nonprescription medical food supplement in Europe since 2008. PEA has been tested in thousands of human subjects without any noted significant side effects. Here we report the first cases of the administration of PEA to two children with autism.* Case Presentations*. The first 13-year-old male child (Subject 1) presented with a total IgE of 572 IU/mL (nl < 200) and with low mature CD57^+^ natural killer cell counts (32 cells/*µ*L; nl = 60–300 cells/*µ*L) and with significant eczema and allergic stigmata. Expressive language, as measured by mean length of utterance, and overall autism severity as measured by the Childhood Autism Rating Scale, Second Edition, improved significantly. Atopic symptoms diminished. No side effects were reported. The second male child, age 15 (Subject 2), also displayed noticeable and rapid improvements in cognitive, behaviors, and sociability.* Conclusion*. Currently, there is no definitive treatment for autism condition. Palmitoylethanolamide could be an effective treatment for autism syndrome. We propose appropriate double-blind clinical trials to further explore palmitoylethanolamide efficacy and safety.

## 1. Introduction

The link between autism spectrum disorders (ASDs) and inflammatory mechanisms both in utero and throughout child development has received extensive support from many sources. Masi et al. recently conducted an extensive review and meta-analysis of the published data on cytokine aberrations in ASDs and found significant evidence for increased interleukin- (IL-) 1beta (*P* < 0.001), IL-6 (*P* = 0.03), IL-8 (*P* = 0.04), interferon-gamma (*P* = 0.02), eotaxin (*P* = 0.01), and monocyte chemotactic protein-1 (*P* < 0.05) [1]. In the same study, they noted the deficit of a counter-regulatory cytokine, transforming growth factor-*β*1. In a detailed case-controlled study, Zerbo et al. also found elevations at birth of the fact that the chemokine monocyte chemotactic protein-1 (MCP-1) predicted future development of ASDs [2]. In a comprehensive review of sixty-seven studies, encompassing nearly 4000 children and adolescents, the evidence for a proinflammatory state was the strongest for ASDs when compared to major depressive disorder (MDD), bipolar disorder (BD), posttraumatic stress disorder (PTSD), obsessive-compulsive disorder (OCD), Tourette's disorder (TD), attention-deficit/hyperactivity disorder (ADHD), and schizophrenia (SZ) [3].

Consistent with these noted inflammatory-state observations in the ASD population are the various immunological interventions published as potential autism therapies. Intravenous immunoglobulin, pentoxifylline, and transfer factor were reviewed by Gupta wherein he noted a significant subpopulation responded to each intervention [4]. Of particular interest to this case was the use of pioglitazone, a thiazolidinedione-class agonist of the peroxisome proliferator activated receptor gamma (PPAR gamma), in an autism population [5]. The authors demonstrated pioglitazone significant reduced irritability, lethargy, stereotypy, and hyperactivity in the children with ASD.

However, pioglitazone significantly may increase the risk of bladder cancer and its use is therefore of limited value to ASDs [7]. The full evaluation of the other and various interventions of potential application of the ASD associated inflammatory state is beyond the scope of this brief report. However, in the search for safer alternatives possessing similar pharmacological effects, palmitoylethanolamide (PEA) emerges as a naturally occurring substance, which has been available as a nonprescription medical food supplement in Europe since 2008 [8]. Nobel laureate Aloe and colleagues initially defined its role as a regulator of mast cells, chronic pain, and inflammation in 1993 [9]. Of interest to ASDs and the large body of literature suggesting part of the chronic inflammatory state maintained in ASDs is mediated via the gastrointestinal system associated immune system [10, 11] (including increased intestinal permeability) [12] is the new evidence describing the anti-inflammatory mechanisms of PEA in gut inflammation in a murine model [13]. The researchers found that exogenous palmitoylethanolamide (intraperitoneally and/or orally) decreased inflammation and improved intestinal permeability, while stimulating colonic cell proliferation and increasing colonic transient receptor potential vanilloid type-1 (TRPV1) and cannabinoid 1 receptor (CB1) expression. The observed anti-inflammatory effect of PEA was attenuated or abrogated by antagonists of the cannabinoid 2 receptor (CB2), the orphan G protein-coupled receptor-55 (GPR55). PPAR antagonists also attenuated the anti-inflammatory effect of PEA.

The potential CNS therapeutic effects of PEA were the subject of a recent and extensive review [14]. The authors note many desirable features of the molecule for chronic brain syndromes. Oral PEA administration has been tested in several thousand human subjects without any noted significant side effects [15, 16]; however, data on its administration to children is limited primarily to treatment or prevention of viral-mediated upper respiratory tract illness [17, 18]. ASDs are also felt to be mediated via complex interactions of microglial cells and mast cells in the central nervous system and macrophages and mast cells in the gastrointestinal system [19]. Recently the interaction between glial cells, mast cells, and the endocannabinoid system (ECS) was the subject of an extensive review [20]. The authors accumulated an abundance of literature supporting the role of mast cell-microglia cross talk in the development of many, if not all, chronic brain inflammatory disorders including autism.

## 2. Case Presentations

### 2.1. Consent

Written informed consent was obtained from the patient's legal guardian(s) for treatment, publication of this case report, and all included data, in compliance with the Code of Ethical Principles for Medical Research Involving Human Subjects of the World Medical Association (Declaration of Helsinki). A copy of the written consent is available for review. All hematological testing was performed as a routine evaluation of the child's immune disorders and was not ordered because of his autism diagnosis.

### 2.2. Subject 1

He is a 13-year-old autistic male with a well-documented history of significant atopic illnesses. He was originally diagnosed with autism by a child neurologist at age of 21 months following a significant regression in child development noted after a viral-like illness with associated high fevers (up to 45.5°C) at age of 15 months. Chronic urticaria, eczema, allergic rhinitis, and asthma have been treated with a variety of antihistamines, montelukast, and topical and systemic steroids since age of 2 years with only temporary beneficial effects. Serum food allergy testing revealed IgE mediated responses to the following: corn, peanut, soybean, wheat, milk, rice, egg, and numerous other dietary proteins. Both skin prick testing and serum IgE inhalant allergy testing revealed similar patterns of significant reactions to most antigens tested including dust mites and nearly all grasses, trees, weeds, molds, and both domestic and farm animals. Both traditional desensitization with injectable antigens and sublingual immunotherapies have failed to reduce allergic stigmata and had no noticeable effects on the course of the child's autism. Total serum IgE testing on numerous occasions has been significantly elevated and at the time PEA was introduced; it was 572 IU/mL (nl ≤ 200). Despite the high total levels of IgE and multiple IgE reactants, blood eosinophils have remained in the normal range throughout. Serum vitamin D-OH25 levels remain deficient despite extensive efforts at oral supplementation, inferring a defect in absorption of fat-soluble nutrients. Serum vitamin D-OH25 levels were at 21 ng/mL at the time PEA was started.

Cellular immune markers reflected specific abnormalities with deficient mature CD57^+^ natural killer (CD57^+^ NK) cell counts 32 cells/*μ*L (nl = 60–300 cells/*μ*L), with a total white count of 6.3 × 10^3^/*μ*L. However, the percentage of lymphocytes was high at 58%, while neutrophils were underrepresented at 33%, and absolute lymphocytes were also elevated at 3.5 × 10^3^ cells/*μ*L. All other obtained typical cell indices were in the normal range.

The Childhood Autism Rating Scale, Second Edition (CARS-2) [21], was administered as part of a routine evaluation of the child's severity prior to a change that is his academic placement. Prior to PEA, CARS-2 scoring placed the child at 43.5 total (82nd percentile) with a verbal communication subscale of 3 out 4. A speech assessment was performed including a mean length of utterance (MLU) measurement. MLU is based on the linguistic concept of morphemes as the smallest component of speech [6]. Speech therapists use MLU as a routine assessment and, in this case, MLU was obtained prior to a change in school placement. MLU was observed to be 3.0 prior to PEA and this corresponded to an age equivalent of approximately 34-35 months (just under a 3-year-old level of speech).

### 2.3. Post-PEA Supplementation (Subject 1)

After the practitioner reviewed the medical literature and the therapeutic rationale with his parents, they treated the child with Normast 600 mg tablets (Epitech Group Srl, Milano, Italy). Normast is available without prescription in Italy and Spain and is classified as “Food for Special Medical Purposes” by the Health Authorities of European Union member states according to standards set forth under European Commission Directive 1999/21/EC.

Initially, 1/2 tablet (300 mg) was given orally with water twice daily on an empty stomach to this boy (a child capable of swallowing tablets). After a week with no observable negative effects, the dose was increased to 1 (600 mg) tablet twice daily. Subject 1's parents returned for assessment after one month of oral supplementation with PEA. They reported “remarkable” changes in his behavior and his expressive language. Specifically, they noted he was spontaneously joining a conversation and commenting on various things happening in the home. The parents also reported the schoolteacher and speech therapist inquired about what had changed and noted similar positive changes at school. Equally noteworthy was the significant reduction in tantrums, outburst, self-talking, and stereotypies. Further, nose-picking, asthmatic cough, allergy stigmata (dark circles under eyes and nasal itching), nasal edema, and both skin eczema and urticaria diminished clinically after a month of PEA administration. No adverse effects were noted.

Following these comments, we readministered the CARS-2 test and noted a total score of 32 (representing the 28th percentile) whereas the starting score was 43.5 (net change 11.5 points and 54 percentiles less). The subscale for expressive language changed to 2 out 4 and represented significant and noticeable changes. Due to these observations, we suggested repeating the MLU assessment for further definition of the degree of language changes. Reassessment of MLU also changed significantly to 5.4 (approximately 58 month's age-equivalency). This represented a 2-year age gain in expressive language in only one month of supplementation with PEA.

Total serum IgE testing after PEA was introduced remained essentially unchanged at 567 IU/mL (nl < 200). Serum vitamin D-OH25 levels rose to 46 ng/mL after PEA was started (possibly due to better absorption). CD57^+^ natural killer (CD57^+^ NK) cell counts increased after PEA to 52 cells/*μ*L (nl = 60–300 cells/*μ*L), with a total white count remaining similar at 5.6 × 10^3^/*μ*L. Noticeably the percentage of lymphocytes reduced to 44%, and the other cell indices were also in the normal range.

### 2.4. Subject 2

He is an Italian 15-year-old male, whom developed normally for the first 2 years, but near 3 years of age, the child developed a progressive loss of previously acquired language, eye contact, and social interaction. Ultimately, this was diagnosed with autism spectrum disorder. At 6 years of age, he experienced his first partial complex epileptic episode, described as the sudden onset of unconsciousness with resultant loss of motor control. The child fell and struck his head although no apparent concussion occurred. He was eventually treated with sodium valproate. The last episode of epilepsy was in March 2006. No recurrent seizures have been reported. After this event the family initiated dietary restriction by eliminating gluten, casein, yeast, sugar, and soy, and they also began supplementation of a multivitamin and additional folic acid. He started diet restriction and vitamins since 2007 at age of 8 years and never stopped. Following these changes the child's overall tone and energy improved and sensory integration also became more appropriate.

The Autism Treatment Evaluation Checklist (ATEC) [22] test was administered as part of a routine evaluation of the child's severity prior to a change in his academic placement. The ATEC has been shown to correlate with the CARS test in domains of sensory/cognitive awareness, speech/language, and sociability [23]. Prior to PEA supplementation the ATEC total scoring was in the mild to moderate range, specifically 25 (a lower number is better): speech 6 of 28 points, sociability 3 of 40 points, sensory/cognitive 1 point (normal), and overall health/behaviors 15 of 75 points. Total serum IgE testing was in the normal range at 127 IU/mL (nl < 200). Serum vitamin D-OH25 levels were normal at 45.10 ng/mL at the time PEA was started. We conducted the observations until three months after treatment. The boy never stopped diet restrictions and multivitamin supplementation since 2007.

### 2.5. PEA Supplementation

**Figure 1:**
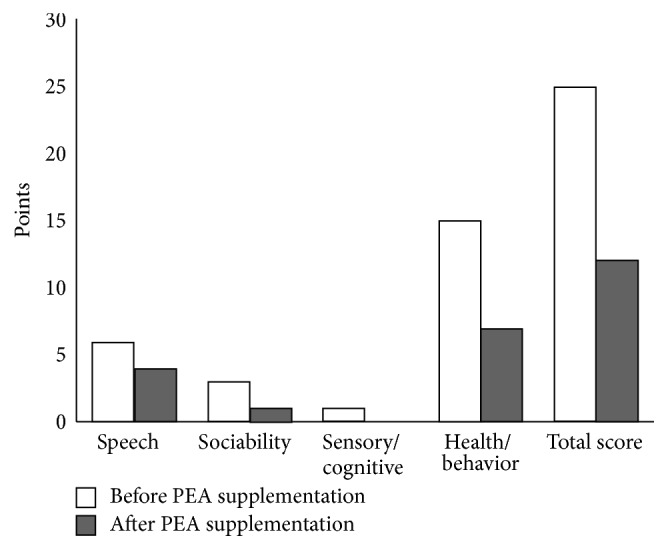
Dynamics on a scale of Autism Treatment Evaluation Checklist (ATEC) in Subject 2 autistic patient before (baseline) PEA supplementation and three months later. The purpose of the ATEC is to measure change in an individual due to various interventions, that is, the difference between the initial (baseline) ATEC scores and later ATEC scores [6].

After practitioner NA reviewed the medical literature and the therapeutic rationale with his parents, they treated the child with Normast 600 mg tablets (Epitech Group Srl, Milano, Italy). After three months of supplementation, ATEC total score reduced by half (12 (after) versus 25 (before) points). The individual scores in the separate domains were speech 4 points, sociability 1 point, sensory/cognitive 0 points, and overall health/behaviors 7 points (Figure 1, Table 1). Also shown for comparison in (Figure 1, Table 1) are the child's improvements in aggression and cognitive and behavioral skills. Language showed mild improvements. In addition, in this case, total serum IgE reduced by half (66.0 post compared to 127 IU/mL pre-PEA). No adverse effects were noted (i.e., nausea, fever, hyperactivity, sleep disorders, skin rash, allergic reactions, and gastrointestinal problems).

## 3. Discussion

Other anti-inflammatory agents beyond those already discussed have been studied in autism. Spironolactone is a potassium sparing diuretic and an anti-inflammatory which downregulates nuclear factor-*κ*B [24]. Bradstreet et al. reported the first case of significant reduction in autism severity and improved expressive language after a short course of spironolactone [25]. Bradstreet et al. also reported favorable responses in children with autism treated with transplantation of fetal stem cells [26]. With similar immunological properties, human cord blood mononuclear cells and umbilical cord-derived mesenchymal stem cells were transplanted into children with autism and again positive benefits were noted [27]. Celecoxib, a cyclooxygenase-2 inhibitor anti-inflammatory, plus risperidone, demonstrated significantly superior effects in irritability than risperidone alone [28].

We have previously shown that circulating monocytes in ASDs express abnormal EC2 activity when compared to controls [29]. PEA may exert its effect on circulating monocyte or directly on mast cells. In the first child's case, the atopy is noteworthy. The previously mentioned research of Aloe et al. [9] directly investigated the mast cell-atopic influences of PEA related chemistry. Of further interest to Subject 1 is the deficiency of mature CD57^+^ NK cells. Atopic dermatitis is associated with decreased circulating NK cells [30]. The author also observed a strong inverse relationship with total IgE and circulating NK cell counts. Wehrmann et al. examined the immunophenotypic characteristics of natural killer (NK) cell subsets in patients with severe atopic dermatitis (AD) and rhinitis allergica (RA) and in healthy controls [31]. As is the case with Subject 1, they found lower percentages of cells with CD57 surface antigens, which correlated with elevations of antigen-specific IgE antibodies to various inhalant allergens.

In neither case did we attempt to measure endocannabinoid levels or PEA in the plasma or cerebral spinal fluid. To our knowledge, commercial laboratories do not offer testing for PEA or other endocannabinoids. Since all of the blood testing was done as a routine part of patient care and not under an investigational protocol, we propose future testing of these levels would be ideal in a research setting. In these particular cases, the parents did not desire a withdrawal of the supplemental PEA because of the ongoing beneficial effects, so we are not able to comment on what might happen with discontinuation of PEA.

Endocannabinoids (EC), including PEA, play an important role in stress and trauma. Individuals with posttraumatic stress disorder (PTSD) had significant alterations of their EC levels, and PEA blood levels were significantly higher in the PTSD population than in controls [32]. In the future, EC- and PEA-commercial blood testing may be a valuable biomarker and a useful tool in predicting and evaluating treatment response to oral supplementation.

One of the concerns regarding oral administration of PEA for neurological therapies is the difficulty of getting the intact PEA past the degradation by intracellular amide hydrolase enzyme activity [33]. This implies the observed effect in these two cases, which may be mediated via its peripheral immune properties in the gastrointestinal tract or through vagal nerve transmission. The galactosyl prodrug of palmitoylethanolamide has been proposed as a superior way to deliver PEA to the CNS [34]. Although this form is not presently available, it may be a target for autism intervention in the future.

## 4. Conclusion

In this original report of two cases, we show PEA-mediated effects may be beneficial for treating core symptoms of autism. PEA is a well-studied, apparently safe even in the pediatric population, anti-inflammatory capable of regulating mast cells and modulating immune chemistry. It is manufactured in the EU and prepared under strict standards according to Good Manufacturing Practice (GMP). PEA also appears to be an atypical endocannabinoid, with additional anti-inflammatory effects mediated via the PPAR pathway. In the United States, recent prevalence data indicate greater than 2% of boys may have ASDs [35, 36]. Given the urgent need for safe and effective strategies for treating ASDs, we propose appropriate double-blind controlled clinical trials to explore further the potential for PEA efficacy and its safety.

## Figures and Tables

**Table 1:** Score distributions on a scale of Autism Treatment Evaluation Checklist (ATEC) in IF autistic patient before (baseline) PEA supplementation and three months later.

	Speech (normal range 0–28)	Sociability (normal range 0–40)	Sensory/cognitive (normal range 0–36)	Behaviors (normal range 0–75)	Total score (normal range 0–180)
Before PEA supplementation	6	3	1	15	25
After PEA supplementation	4	1	0	7	12

## Abbreviations

AD: Atopic dermatitis
ADHD: Attention-deficit/hyperactivity disorder
ASDs: Autism spectrum disorders
ATEC: Autism treatment evaluation checklist
BP: Bipolar disorder
CB: Cannabinoid receptor
CARS-2: Childhood autism rating scale, second edition
CNS: Central nervous system
EC: Endocannabinoids
ECS: Endocannabinoid system
GMP: Good manufacturing practice
GPR55: G protein-coupled receptor-55
Ig: Immunoglobulin
IL: Interleukin
IU: International unit
MDD: Major depressive disorder
MLU: Mean length of utterance
NK: Natural killer
OCD: Obsessive-compulsive disorder
PEA: Palmitoylethanolamide
PPAR: Peroxisome proliferator activated receptor
PTSD: Posttraumatic stress disorder
RA: Rhinitis allergica
SZ: Schizophrenia
TD: Tourette's disorder
TRPV1: Transient receptor potential vanilloid type-1.
